# Laparoscopic duodenum-preserving pancreatic head resection: A case report

**DOI:** 10.1097/MD.0000000000004442

**Published:** 2016-08-12

**Authors:** Jiayu Zhou, Yucheng Zhou, Yiping Mou, Tao Xia, Xiaowu Xu, Weiwei Jin, Renchao Zhang, Chao Lu, Ronggao Chen

**Affiliations:** a Department of General Surgery, School of Medicine, Zhejiang University, Hangzhou, Zhejiang Province, China; b Department of General Surgery, Zhejiang Provincial People's Hospital, Hangzhou, Zhejiang Province, China.

**Keywords:** duodenum-preserving pancreatic head resection, minimally invasive surgery, SPN

## Abstract

**Background:**

Solid pseudopapillary neoplasms (SPNs) of the pancreas are uncommon neoplasms and are potentially malignant. Complete resection is advised due to rare recurrence and metastasis. Duodenum-preserving pancreatic head resection (DPPHR) is indicated for SPNs located in the pancreatic head and is only performed using the open approach. To the best of our knowledge, there are no reports describing laparoscopic DPPHR (LDPPHR) for SPNs.

**Methods:**

Herein, we report a case of 41-year-old female presented with a 1-week history of epigastric abdominal discomfort, and founded an SPN of the pancreatic head by abdominal computed tomography/magnetic resonance, who was treated by radical LDPPHR without complications, such as pancreatic fistula and bile leakage. Histological examination of the resected specimen confirmed the diagnosis of SPN.

**Results:**

The patient was discharged 1 week after surgery following an uneventful postoperative period. She was followed up 3 months without readmission and local recurrence according to abdominal ultrasound.

**Conclusion:**

LDPPHR is a safe, feasible, and effective surgical procedure for SPNs.

## 1 Introduction

In the 1970s, Beger et al ^[1]^ developed duodenum-preserving pancreatic head resection (DPPHR) for patients with an inflammatory mass in the head of the pancreas caused by chronic pancreatitis. Compared with pancreaticoduodenectomy, the Beger procedure maintained the integrity of the duodenum and biliary tract. With the advantage of organ preservation, this procedure has evoked worldwide attention. Following several years of study and practice, more and more pancreatic surgeons are now performing DPPHR to remove benign or low-grade malignant tumors in the pancreatic head, such as solid pseudopapillary neoplasms (SPNs), and cystic neoplasms. [^2^ ^3^ ^4^]

With the advances in techniques, minimally invasive technology has become a mainstream method for abdominal diseases. ^[5]^ Owing to the complex anatomy and rich blood supply of the pancreas, progress has been slow in practice of laparoscopic pancreatic surgery, especially laparoscopic duodenum-preserving pancreatic head resection (LDPPHR). Until now, there is no report describing this surgical procedure. We here report a case of 41-year-old female with an SPN of the pancreatic head, who was treated with LDPPHR as a novel minimally invasive surgery.

## 2 Case report of LDPPHR

**Figure 1:**
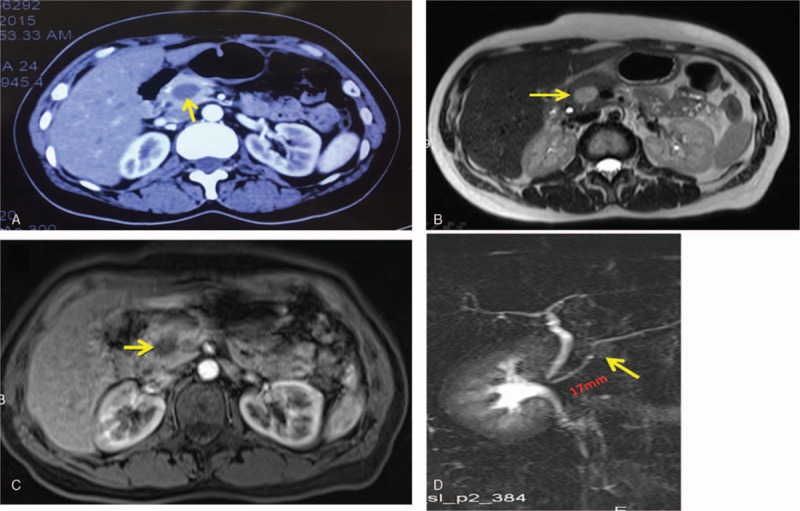
CT, MRI, and MRCP scans. (A) Contrast-enhanced CT and (B, C) contrast-enhanced MRI showed a mixed density tumor in the head of the pancreas (yellow arrow). (D) MRCP showed the main pancreatic duct without dilation, approximately 1.7 cm between the mass (yellow arrow) and Vater's ampulla. CT = computed tomography, MRI = magnetic resonance imaging; MRCP = magnetic resonance cholangiopancreatography.

A 41-year-old female was admitted to our hospital on October 9, 2015 with a 1-week history of epigastric abdominal discomfort. There were no significant findings on physical examination. Her past medical history was unremarkable, with the exception of an allergy to penicillin. Tumor markers including carbohydrate antigen (CA)19-9, carcinoembryonic antigen(CEA), CA72-4, and CA12-5 were all normal. Other laboratory findings such as serum levels of total bilirubin, amylase, and immunoglobulin G (IgG)-4 were within the normal range. Abdominal contrast-enhanced computed tomography (CT) scan revealed a 2.2 × 1.7 cm cystic-solid mass showing inhomogeneous enhancement in the head of the pancreas (Fig. 1A). Magnetic resonance (MR) imaging and MR cholangiopancreatography confirmed the presence of a mass in the pancreatic head without pancreatic duct and common bile duct (CBD) dilation (Fig. 1B, C). The distance between the tumor and Vater's ampulla was approximately 1.7 cm (Fig. 1D).

**Figure 2:**
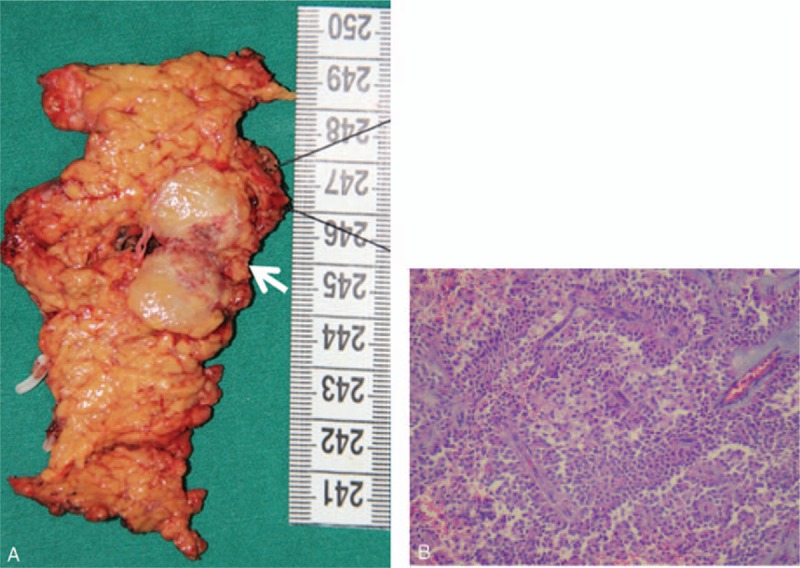
(A) Gross appearance of the SPN. A well-capsulated mass, 2.2 × 1.7 cm in size, was located in the pancreatic neck (white arrowhead). (B) Histopathology of the SPN (H&E × 100). The tumor showed papillary structures with cyst-like spaces. H&E = hematoxylin & eosin, SPN = solid pseudopapillary neoplasm.

After preoperative examinations, the informed consent was signed and then LDPPHR was performed. Intraoperative frozen section further confirmed SPN, and R0 resection was achieved. Operation time was 210 minutes, and blood loss was 150 mL. On the first postoperative day (POD1), the patient achieved out-of-bed activity. She started a liquid diet on POD3 and was discharged on POD7. No pancreatic fistula or bile leakage was found during the hospital stay. Histopathological and immunohistochemical examination of the resected specimen revealed that the SPN was Neuron-specific enolase (NSE)-positive, Synaptophysin (SYN)-positive, Chromogranin A (CgA)-negative, Cluster of Differentiation (CD10)-positive, Alpha-1 antitrypsin chymotrypsin (AACT)-positive, and Alpha-1 antitrypsin (AAT)-positive (Fig. 2A, B). Three months after operation, the patient was followed up and no any discomfort was founded after taking solid foods. Meanwhile, laboratory examinations including blood routine examination, amylase, and biochemical tests were normal. Abdominal ultrasound confirmed local recurrence.

**Figure 3:**
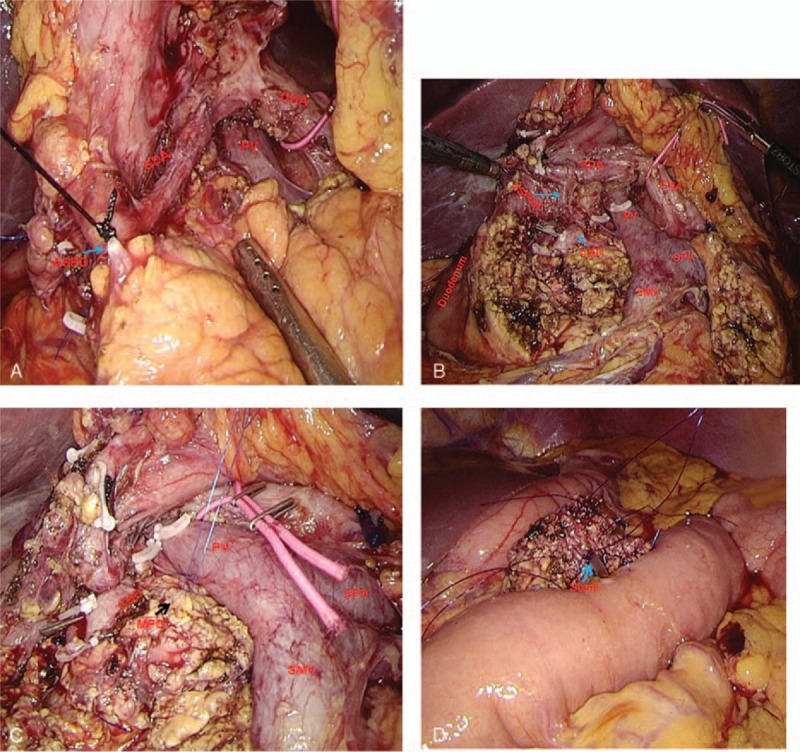
Photograghs of the laparoscopic duodenum-preserving pancreatic head resection (LDPPHR) procedure: (A) exposing the gastroduodenal artery (GDA) and excising the anterior superior pancreatoduodenal artery (ASPD); (B) status after complete resection of the pancreatic head; (C) exposure of the main pancreatic duct (MPD); and (D) reconstruction. The pancreaticojejunostomy was between the neck of the pancreas and the jejunal loop (duct-to-mucosa, blue arrow).

The surgical procedure, a “bottom-up” sequence, was as follows (Fig. 3): placing 5 trocars in a V-shape; dividing the gastrocolic ligament, exposing the pancreas, and excising the anterosuperior pancreatoduodenal artery; establishing a portal vein tunnel; determining the tumor location using intraoperative laparoscopic ultrasound; resecting the pancreatic head containing the tumor along the duodenum using a harmonic scalpel while preserving the main arcade of the posterior pancreaticoduodenal artery; intraoperative frozen section confirmed an SPN with a negative margin; and closing the proximal end of the main pancreatic duct (MPD), and performing an end-to-side pancreaticojejunostomy (duct-to-mucosa).

## 3 Discussion

SPN is a rare pancreatic tumor, without obvious symptoms, that usually affects young females, and accounts for approximately 1% to 2% of all exocrine pancreatic neoplasms.[ ^6^ ^7^] In 2010, SPN was classified as a low-grade malignant neoplasm with an excellent prognosis by the WHO. ^[8]^ Therefore, complete en-bloc resection is recommended in the absence of distant metastasis. However, there is controversy regarding the available surgical treatments for SPNs in the pancreatic head, which include DPPHR, pylorus-preserving pancreaticoduodenectomy, and pancreaticoduodenectomy.

In our case, we performed LDPPHR for SPN of the pancreatic head. The following 3 factors were taken into consideration in our choice of surgery.

- The location, size, and nature of the pancreatic tumor are crucial in making this decision. Preoperative imaging is useful for diagnosing SPN, although the diagnostic accuracy for SPN is only 82%. ^[9]^ According to the abdominal CT and MRI findings in our case, we identified a typical SPN located in the pancreatic head, 3 × 3 cm in size, which was an encapsulated round mass containing solid, necrotic, and hemorrhagic components. Furthermore, as previously mentioned, the distance between the tumor and Vater's ampulla was >1 cm. No distant metastasis or local lymph node involvement were detected. Therefore, LDPPHR was performed in accordance with current studies which suggested that DPPHR is indicated for SPNs in the pancreatic head and prophylactic lymph node dissection is not warranted. ^[10]^ Intraoperative ultrasound and frozen section further confirmed the diagnosis and ascertained the resection margins.
- DPPHR is a less traumatic organ-preserving surgical procedure. A meta-analysis by Sukharamwala ^[11]^ proved the superiority of DPPHR compared with pancreaticoduodenectomy for benign or low-malignant tumors with regard to lower postoperative morbidity. With the development of laparoscopic pancreatic surgery, the minimally invasive method of LDPPHR was introduced. LDPPHR represents the precision of minimally invasive surgery. This technique results in not only organ preservation, but also rapid postoperative recovery. Our patient developed no pancreatic fistula according to the International Study Group of Pancreatic Fistula, and no other complications such as bile leakage or duodenal ischemia.
- LDPPHR is technically feasible at our center. It is known that laparoscopic surgery of the pancreas has developed slowly, mainly due to the complex anatomy, rich blood supply, and critical surgical skills. ^[12]^ Between March 2013 and June 2015, we successfully performed laparoscopic pancreatoduodenectomy in 120 cases using the 5-hole approach. Although there are no published reports on LDPPHR, we have completed this complex surgery based on our experience.

Although open and LDPPHR involve different operative pathways, the key surgical points are the same and are as follows: to ensure the blood supply in the duodenum and bile duct, Takada et al ^[13]^ demonstrated that the posterosuperior pancreatoduodenal artery is important to avoid ischemia. In our case, retropancreatic fascia and fibrotic tissue along the CBD were preserved to ensure the blood supply. Avoid injury to the intrapancreatic CBD. Two common reasons contributing to injury are the difficulties in identifying the pancreatic segment of the CBD and the use of a hook. Therefore, an ultrasonically activated scalpel was used to bluntly dissect the pancreatic tissue step by step. The bile duct was thus identified easily. Prevention of intraoperative pancreatic fistula. As reported in Beger's studies,[ ^14^ ^15^] the incidence of pancreatic fistula was high. In our case, pancreatic fistula did not occur. Four factors, such as reliable pancreaticojejunostomy, definite suture of the duodenal side of the MPD, embedding of the residual pancreatic tissue near to the duodenum, and coagulation of the ultrasonically activated scalpel, may have prevented this complication.

In conclusion, our study indicates that LDPPHR for the treatment of SPN not only resulted in complete resection of the pancreatic tumor, but also contributed to rapid postoperative recovery. However, the long-term outcome of this procedure needs to be confirmed by the completion of more clinical trials.
